# SCRMS: An RFID and Sensor Web-Enabled Smart Cultural Relics Management System

**DOI:** 10.3390/s17010060

**Published:** 2016-12-30

**Authors:** Changjiang Xiao, Nengcheng Chen, Dandan Li, You Lv, Jianya Gong

**Affiliations:** 1 State Key Lab for Information Engineering in Surveying, Mapping and Remote Sensing, Wuhan University, 129 Luoyu Road, Wuhan 430079, China; cjxiao@whu.edu.cn (C.X.); yorgurts@whu.edu.cn (D.L.); lvyou@whu.edu.cn (Y.L.); gongjy@whu.edu.cn (J.G.); 2 Collaborative Innovation Center of Geospatial Technology, 129 Luoyu Road, Wuhan 430079, China; 3 School of Remote Sensing and Information Engineering, 129 Luoyu Road, Wuhan 430079, China

**Keywords:** smart city, cultural relics, Internet of Things (IoT), museum, RFID, Sensor Web, multiple sensing, smart monitoring

## Abstract

Cultural relics represent national or even global resources of inestimable value. How to efficiently manage and preserve these cultural relics is a vitally important issue. To achieve this goal, this study proposed, designed, and implemented an RFID and Sensor Web–enabled smart cultural relics management system (SCRMS). In this system, active photovoltaic subtle energy-powered Radio Frequency Identification (RFID) is used for long-range contactless identification and lifecycle management of cultural relics during their storage and circulation. In addition, different types of ambient sensors are integrated with the RFID tags and deployed around cultural relics to monitor their environmental parameters, helping to ensure that they remain in good condition. An Android-based smart mobile application, as middleware, is used in collaboration with RFID readers to collect information and provide convenient management for the circulation of cultural relics. Moreover, multiple sensing techniques are taken advantage of simultaneously for preservation of cultural relics. The proposed system was successfully applied to a museum in the Yongding District, Fujian Province, China, demonstrating its feasibility and advantages for smart and efficient management and preservation of cultural relics.

## 1. Introduction

Cultural relics represent national or even global resources of inestimable value [1]. Relics are a type of historic and cultural inheritance that are usually used for enjoyment and educational purposes. Thus relic management and preservation is of vital importance. Relics can be found stored in warehouses, exhibited in museums, and sometimes circulated among museums for various purposes. During these processes, two issues are fairly critical: (1) massive numbers of cultural relics must be identified and recorded as they move in and out of museums and also checked frequently for stock inventory purposes; (2) damage from the natural environment, thefts and lost items occur frequently, causing serious cultural and socio-economic losses. For the first issue, many museums still adopt traditional schemes, including backward manual handwritten methods which are quite time consuming and error-prone, and barcode methods that require line-of-sight of the cultural relics. Therefore, there is a strong desire for intelligent identification and management systems that can both improve the efficiency and reduce errors. For the second issue, traditional methods primarily rely on individual human inspection or video surveillance. However, these approaches involve considerable human labor and do not achieve satisfactory results. Thus, there is an urgent need for an intelligent, efficient and effective means to manage and safeguard these precious cultural relics.

Radio Frequency Identification (RFID), one of the core technologies of the Internet of Things which is considered as the future evolution of the Internet [2,3], can be used to uniquely identify objects in the physical world. Unlike earlier bar-code technology, it does so without requiring a line of sight. Furthermore, RFID systems can discern between many tags located in the same general area without human assistance [4]. The Sensor Web, first proposed by Delin and Jackson [5], is considered as a smart macro instrument to raise awareness of the environment context, during which various communication technologies (e.g., WiFi, 3G, 4G, and WiMax) are used to communicate the real-time status of the real world [6,7].

Due to the excellent features in efficient object identification, ubiquitous sensing and indoor localization, RFID has been widely used for supply-chain management, smart tourism, smart grids and so forth [8,9,10,11]. Specifically, in the cultural heritage domain, RFID has been used for audits and stock-taking of museum collections [12,13], as well as enhanced user experience in heritage exhibitions [14]. RFID has also been further used with the Sensor Web to fully take advantage of the strengths of both technologies. For example, Chianese et al. designed a location-based smart application called SmARTweet for exhibitions and museums using RFID and sensor technologies. In this approach, sensors were deployed to allow visitors’ mobile devices to detect the closest artwork and thus provide personalized content [15,16]. Further, they took advantage of RFID and sensors to capture context information and detect events in their proposed smart Context Evolution System (CES), which can dynamically provide useful data and services to users in accordance with context switches [17]. Xing et al. proposed an RFID-based approach to monitor treasures to determine whether they have been changed. In this approach, the treasures are equipped with sensor nodes and wireless communication equipment [18].

From the studies mentioned above, it is apparent that RFID and Sensor Web technologies have shown great suitability and advantages for indoor smart applications closely related to location, environmental parameters and similar contexts. Nevertheless, in the cultural heritage domain, most of the attention has been paid to enhancing the user experience in exhibitions. Though there are some solutions using RFID for museum art collection inventory, the preservation of cultural relics from environmental or artificial damages—tasks that are both fundamental and vitally important, is still not satisfactory. Thus, in this paper, considering the suitability of RFID and Sensor Web technologies and their current level of development for applications in the cultural heritage domain, we design and develop an RFID and Sensor Web-enabled system for managing and preserving cultural relics in an intelligent and efficient manner, which provides a new perspective on solving the aforementioned problems. This study makes the following three contributions:

(1) The system offers efficient and intelligent management for cultural relics. It takes advantage of RFID, Android-based and web-based applications to collaboratively and automatically identify, conveniently register and efficiently manage information concerning cultural relics, improving the degree of intelligence and reducing the time needed and errors made.
(2) The system provides safe, efficient protection for cultural relics. In the proposed system, RFID technology is innovatively used to detect whether the targeted cultural relics are within a predefined safe range. Further, the RFID, sensors and video surveillance are collaboratively used to improve the effects of protection of cultural relics from both environmental and artificial damages, as well as thefts. Rules were designed to determine whether it should alarm to initiate further actions based on sensing results of these three types of techniques.
(3) A demo application of the system was tested in a real-world scenario: a museum located in Yongding District, Fujian Province, China. In this demonstration, several types of ambient sensors, RFID, an Android-based terminal application and a server-side web application were adopted and deployed. It successfully tested the feasibility and suitability of the proposed system for managing and safeguarding cultural relics.

The reminder of this paper is organized as follows: Section 2 and Section 3 present the design and implementation of the proposed system, respectively. In Section 4, we discuss features of the proposed system based on the demonstration application discussed in Section 3. Finally, we provide conclusions and some directions for future work in Section 5.

## 2. System Design

### 2.1. RFID and Sensor Web-based System Architecture

**Figure 1:**
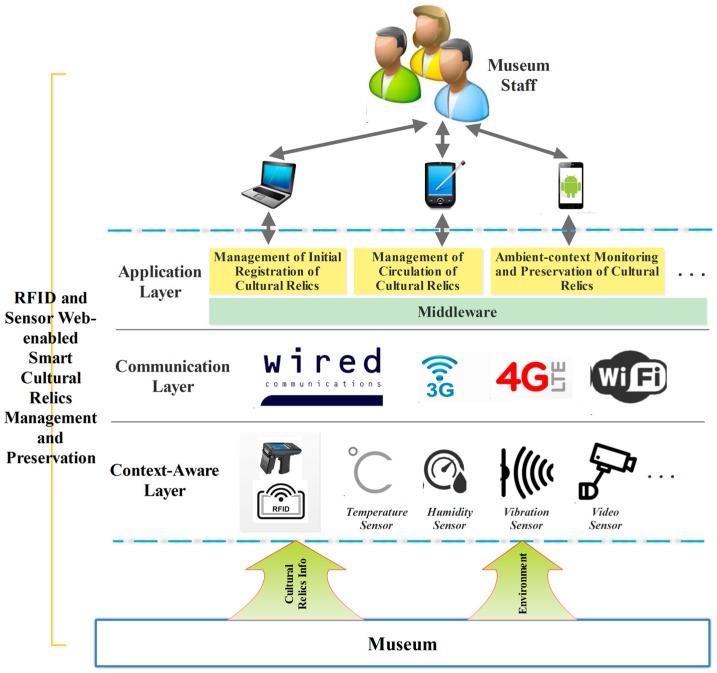
Architecture of the proposed system.

Considering the requirements for managing and preservation of cultural relics mentioned in Section 1, the architecture of the proposed system is designed with three layers, including a context-aware layer, a communication layer and an application layer, as depicted in Figure 1. The system is described in more detail in the following subsections.

#### 2.1.1. Context-Aware Layer

The context-aware layer is the fundamental layer of the whole architecture. It consists of physical objects and sensor devices [2]. The physical objects in the museum scenario are primarily cultural relics, exhibition rooms, warehouses and so forth. The sensor devices include RFID tags, temperature sensors, humidity sensors, vibration sensors, video sensors, and so on. This layer is responsible for the identification of cultural relics and for collecting the context information specific to the cultural relics. Information collected in this layer is then transmitted to the application layer through proprietary communication manners in the communication layer.

#### 2.1.2. Communication Layer

The communication layer is responsible for transmitting information collected in the context-aware layer to the application layer, or more specifically, the middleware sub-layer of the application layer. The means of communication include both wired and wireless transmissions. The wireless transmissions further consist of 3G, 4G, WiFi, Bluetooth, ZigBee and so forth. Adopting which communication means depends on sensor devices and communication constraints.

#### 2.1.3. Application Layer

The application layer is actually comprised of two sub-layers, namely, a middleware sub-layer and a cultural relics management-oriented applications sub-layer. The middleware sub-layer is responsible for receiving raw data from the sensors in the context-aware layer and transforming the received data into a form that can be understood by human beings according to predefined communication protocols. It is also responsible for sending the transformed data to back-end servers to support higher-level applications. The cultural relic management-oriented applications sub-layer is primarily comprised of applications for managing relics' transitions in and out of the museum, inventory, and preservation. These applications operate on different platforms and devices, depending on their implementation mechanisms and their roles and functions in the entire architecture.

### 2.2. Cultural Relics-Centric System Modeling

**Figure 2:**
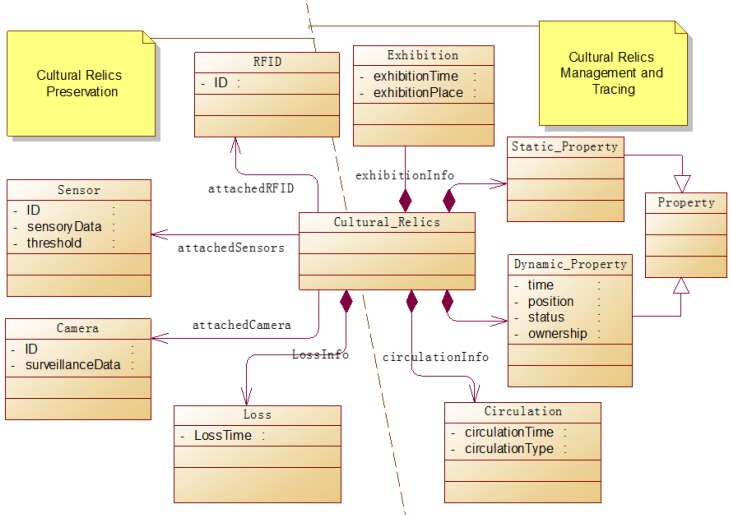
Cultural relics-centric system modeling.

The goal of the proposed system is to both manage the cultural relics and preserve them from damage and loss. Figure 2 shows a cultural relics-centric model of the system. There are primarily four types of objects in this system: cultural relics, RFID tags, ambient sensors, and cameras. RFID tags are attached to cultural relics for their unique identification, while ambient sensors and cameras are deployed and associated with cultural relics for real-time monitoring and protection. They produce real-time sensory data and surveillance data, revealing the dynamics of the environment around cultural relics. Cultural relics are exhibited in certain places during certain periods of time and are sometimes circulated among museums for some purposes. During such movements, their positions are changed, and sometimes their status and ownership are changed as well. Such information is modeled as dynamic properties of cultural relics, recording spatio-temporal track of cultural relics which can be used for tracing them.

### 2.3. Logical Data Model

**Figure 3:**
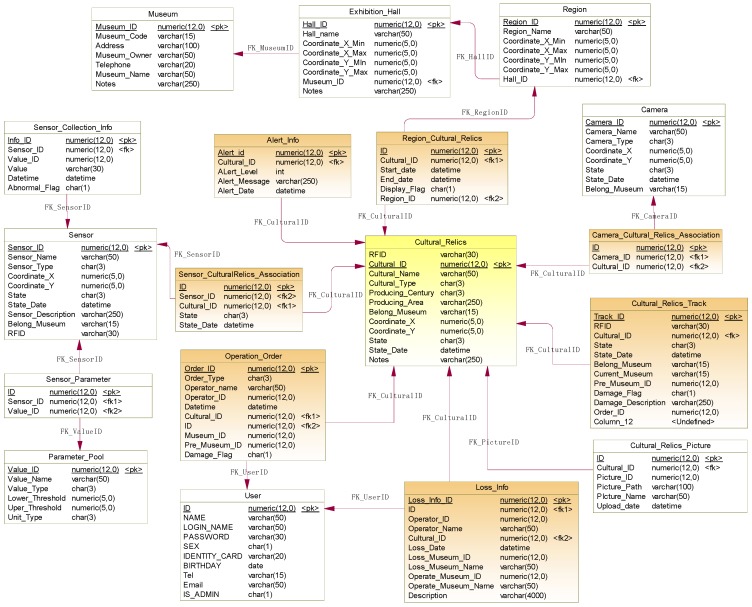
Logical data model of the proposed system.

Based on the system modeling results depicted above, business process analysis and system function requirement analysis, we designed logical data model of the system that depicts and store both static and dynamic information, and relations between objects in the SCRMS. A diagram of this data model is shown in Figure 3, consisting of 18 tables and relations between them. These tables can be divided into the following eight categories from the perspective of their roles in the process.

- The cultural relics-related tables include *Cultural_Relics* and *Cultural_Relics_Picture*. These tables are primarily used to store the basic metadata of cultural relics that is collected when they are first registered or subsequently updated. Specifically, RFID is associated with cultural relics through the field *RFID* (a unique ID) in the *Cultural_Relics* table.
- The exhibition-related tables include *Museum*, *Exhibition_Hall*, *Region* and *Region_Cultural_Relics*. From these tables, the hierarchical exhibition space from museum to exhibition hall, and then from exhibition hall to region, is expressed. The spatial coordinates of each exhibition space are measured and recorded in the corresponding tables. Using these coordinates, we can easily locate a specific cultural relic. Further, inquiry of cultural relics based on spatial filters can be realized.
- The circulation-related tables include *Operation_Order* and *Cultural_Relics_Track*. *Operation_Order* stores the details of every circulation (in-out of the museum) of cultural relics, which is critical for further check and inventory. This information is uploaded through mobile terminals at every circulation operation. The *Cultural_Relics_Track* table records the tracking information for cultural relics to accompany their circulation (i.e., where it is now and where it was last). This information is useful in preserving cultural relics.
- The sensor-related tables, including *Sensor_Collection_Info*, *Sensor*, *Sensor_Parameter*, and *Sensor_CulturalRelics_Association*. They store both static and dynamic (i.e., sensor status) sensor metadata and dynamic sensory data, and meanwhile reveal the association relation between sensors and the cultural relics they monitor.
- The camera-related tables, including *Camera* and *Camera_Cultural_Relics_Association*, with the first recording camera metadata and the second revealing the association relation between the cultural relics and the cameras that monitor them.
- The abnormality- and alert-related tables include *Loss_Info* and *Alert_Info*. Specifically, *Loss_Info* records loss info of cultural relics, including which ones have been lost and when and where they were lost and so on. The *Alert_Info* table stores all the alerts related to abnormal ambient contexts and safety issues of the cultural relics in predefined formats.
- The user-related table (*User*) stores basic and required information about users of the system. It can be used for administration and authentication of both user and system functions. In the proposed system, a user account must be allocated by system administrator; users cannot self-register for security considerations.
- The system configuration-related table is called *Parameter_Pool*. This table stores metadata of all the parameters in the proposed system, which is helpful for plug and play of sensors and cameras.

### 2.4. Communication Interfaces

**Figure 4:**
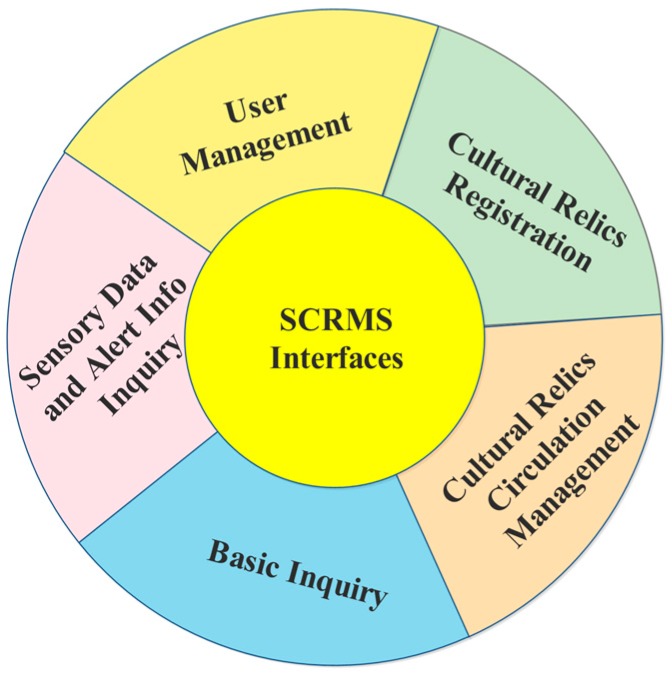
Five categories of interfaces.

To achieve the targeted management of cultural relics, five categories of communication interfaces are designed based on system functions, as illustrated in Figure 4. Input and output parameters of the interfaces are all in the form of JavaScript Object Notation (JSON) [19], which is a lightweight data-interchange format, easy for humans to read and write, and easy for machine to parse and generate, reducing volume of data transferred between clients and servers. Using these interfaces, the system becomes loosely coupled and easy to extend and maintain.

- Interfaces for management of user information: These interfaces are primarily used for creation and authentication of system users, verification of user authentication and edition of user information.
- Interface for initial registration of cultural relics: Registration information includes unique ID of the RFID tag attached to cultural relics, metadata of cultural relics (e.g., name, type, dynasty, excavation place, belonging museum, exhibition hall, and images etc.), attached sensors and camera information, and any optional notes.
- Interfaces for management of circulation information of cultural relics: One is *CR_in_register,* which is used to register all the cultural relics that enter the museum, and the other is *CR_out_register* which is used to register cultural relics that leave the museum. Parameters both interfaces require are: a timestamp of the registration operation, damage flag (as detailed in Equation (1)), ID of the staff member handling the registration, list of IDs of cultural relics that are moving into or out of the museum, and optional notes. There are also two non-shared parameters: (1) in *CR_in_register*, the *preMuseumID* parameter indicates the museum a cultural relic arrived from, and an *in*_*order_type* indicating the purpose of entry, as expressed in Equation (2); (2) in *CR_out_register*, the *targtedMuseumID* parameter indicates the museum to which a cultural relic is going, and an *out_order_type* that indicates the purpose of the move, as expressed in Equation (3):
  (1) $$\mathrm{damageFlag}=\left\{ \begin{array}{l} 0\;\mathrm{if}\;\mathrm{cultural}\;\mathrm{relics}\;\mathrm{is}\;\mathrm{normal}\;\mathrm{and}\;\mathrm{in}\;\mathrm{good}\;\mathrm{condition}\\ 1\;\mathrm{otherwise} \end{array}\right.,$$
  (2) $$\mathrm{in}\text{\_}\mathrm{order}\text{\_}\mathrm{type}=\left\{ \begin{array}{l} 001\;\mathrm{lent}\;\mathrm{before}\;\mathrm{and}\;\mathrm{now}\;\mathrm{returned}\\ 002\;\mathrm{borrowed}\;\mathrm{for}\;\mathrm{display}\\ 003\;\mathrm{immigrated}\;\mathrm{from}\;\mathrm{other}\;\mathrm{museum} \end{array}\right.,$$
  (3) $$\mathrm{out}\text{\_}\mathrm{order}\text{\_}\mathrm{type}=\left\{ \begin{array}{l} 101\;\mathrm{lent}\;\mathrm{for}\;\mathrm{exhibition}\\ 102\;\mathrm{emigrated}\;\mathrm{to}\;\mathrm{another}\;\mathrm{museum}\\ 103\;\mathrm{scrap}\;\mathrm{registration} \end{array}\right.,$$
- Interfaces for inquiries of basic information These interfaces are designed to query the basic information of cultural relics, sensors, and cameras according to predefined filters, such as acquiring a list of cultural relics whose status are normal or obtaining sensor information (value type, lower threshold and upper threshold of measures between which the cultural relics can remain in good condition) based on sensor ID and so forth.
- Interfaces for inquiry of sensory data and alert information: Sensory data are obtained based on sensor ID and spatio-temporal constraints, while alert information is obtained through temporal filters.

## 3. System Implementation and Case Study

### 3.1. System Components

#### 3.1.1. Context-Aware Hardware 

**Figure 5:**
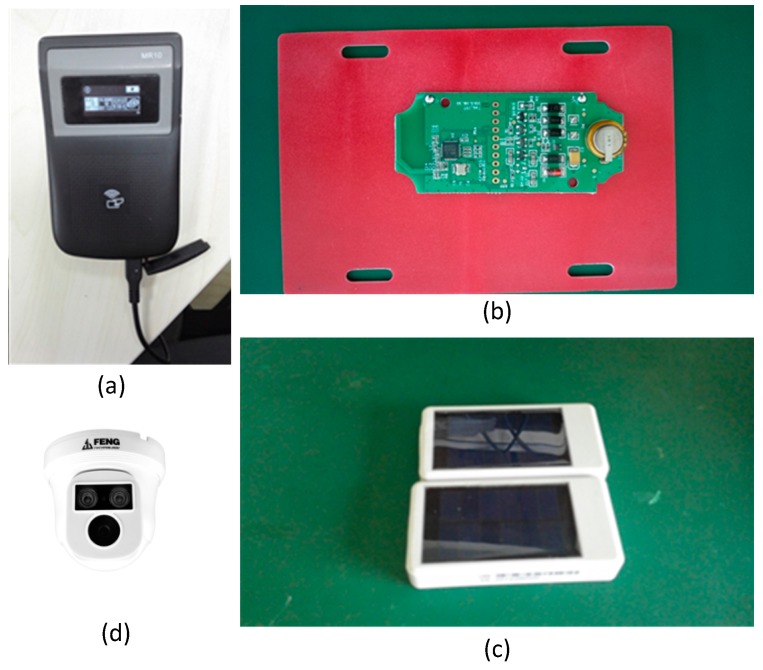
Context-aware hardware: (**a**) RFID reader; (**b**) Integrated circuit board containing RFID tag and ambient sensors; (**c**) The integrated circuit board is placed in a box to prevent potential damage from dust, water and other factors; (**d**) High-definition video cameras for real-time surveillance of cultural relics with infrared imaging and motion detection features.

In the proposed system, four categories of context-aware hardware are adopted: (1) RFID tags and readers (as shown in Figure 5a) are used to bind each cultural relic to a specific RFID tag at initial registration, to identify cultural relics through the attached RFID tag, and to acquire details from or upload circulation information of cultural relics to back-end server based on the unique ID stored in the RFID tag; (2) Ambient sensors in the proposed system include sensors for collecting air temperature, air humidity, vibration and displacement of cultural relics; (3) Video cameras are deployed in collaboration with the RFID tag and vibration sensor for security monitoring purposes to help prevent theft; (4) Mobile terminals are used by museum staff to perform registration and circulation-related operations, using a developed mobile client application installed on the mobile terminals. Specifications of the three categories of hardware are detailed in Table 1.

In our proposed system, the RFID tag and ambient sensors are integrated into one small circuit board (as shown in Figure 5b), facilitating the deployment and integrated sensing of ambient context. The board is then placed in a box to prevent dust, water and other potential damage factors, as shown in Figure 5c. Video cameras, as shown in Figure 5d, can monitor exhibition halls and cultural relics in real time, with features of infrared imaging, motion detection and motion states prediction [20] which can aid in theft prevention during both day and night.

Data is transmitted using both wired and wireless communications: video data is wired, while RFID data is Bluetooth and the ambient sensors use GPRS.

#### 3.1.2. Data Collection and Administration Software

**Figure 6:**
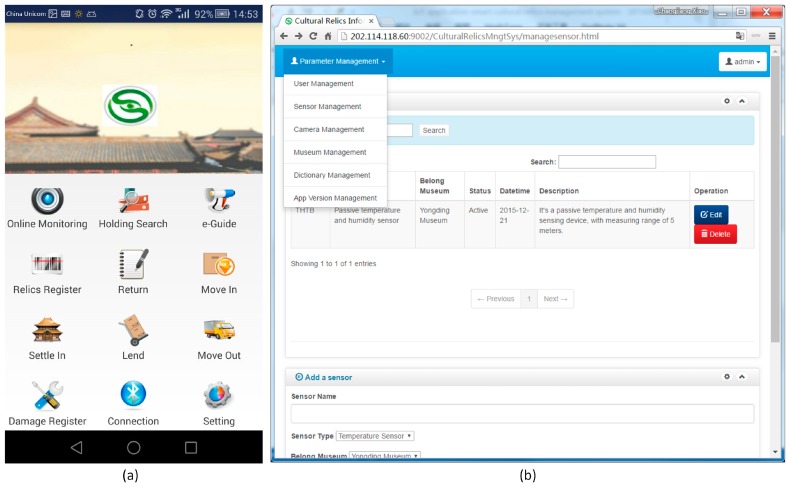
Data collection and administration software: (**a**) Android-based middleware and application; (**b**) Server-side cultural relics information management system.

In the proposed system, three types of software are developed for data collection and administration, operating on different platforms:

- Android-based middleware and application. The reason why it is called middleware is that it can obtain RFID data by communicating with an RFID reader via Bluetooth, and further uploads the collected RFID data to a back-end server. It’s also a client application, which provides well-designed graphical user interfaces (GUI) for staff in museum to conduct management tasks for cultural relics including registration and circulation-related tasks. The main GUI is illustrated in Figure 6a.

- Windows service-based ambient data collection middleware. This middleware is implemented as a kind of Windows service, without GUIs, which runs in the background silently and can reduce consumption of system resources. It communicates with the ambient sensors through GPRS connections, sends data requests periodically, parses the received data according to a predefined protocol, and stores parsed sensory data to a back-end MySQL database management system.
- Web-based server-side cultural relics information management system. It’s implemented based on the open source Struts2 framework [21,22]. The web clients are based on Bootstrap, which is the most popular HTML, Cascading Style Sheet(CSS) and JavaScript (JS) framework for developing responsive projects on the web [23], and jQuery [24,25]. This server-side system provides user management, sensor management, video cameras management, cultural relics management, as well as Android application version management functions to system administrators through a web browser, contributing to convenient and flexible management wherever the Internet is accessible, as illustrated in Figure 6b.

### 3.2. Key Technologies

#### 3.2.1. RFID-Based Identification and Management of Cultural Relics

**Figure 7:**
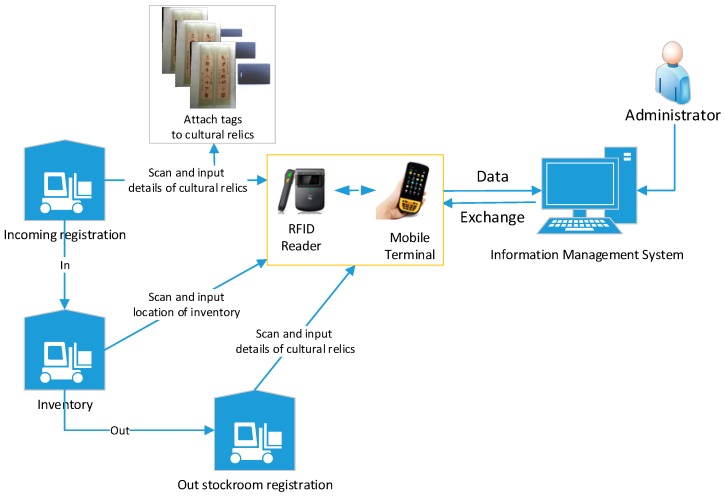
RFID-based identification and management of cultural relics.

In our proposed system, to realize smart and efficient management, all cultural relics are attached with an RFID tag each, which stores a unique ID, at their first registration, as shown in Figure 7. Operators can then easily upload and register the details of these cultural relics to the server-side information management system through the Android-based application. These information is stored in a back-end server-side database, serving as a profile of the virtual personalities of each cultural relic indexed by the unique ID. When cultural relics enter the museum or are shipped to another museum, operators simply scan the RFID tags attached to them and register corresponding entry/exit information in the information management system using the aforementioned Android application.

#### 3.2.2. Collaborative Preservation of Cultural Relics

**Figure 8:**
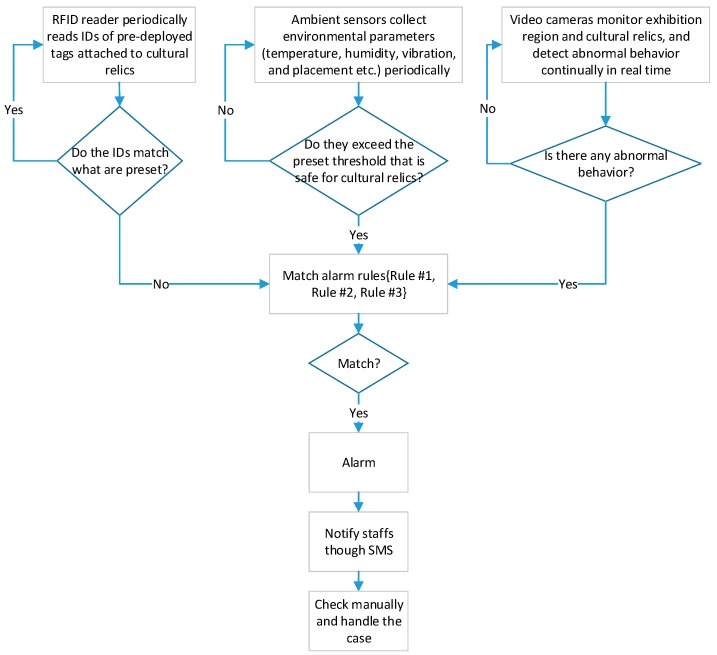
Flowchart of collaborative loss prevention for cultural relics.

**Figure 9:**
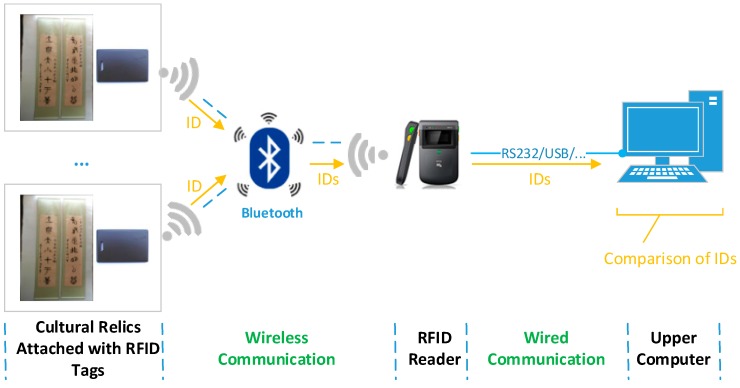
RFID-based loss prevention for cultural relics.

Traditional methods often adopt singular preservation scheme, which leads to low accuracy. To overcome this limitation, the proposed system collaboratively uses multiple sensing techniques to safeguard cultural relics. As shown in Figure 8, RFID technology, Sensor Web technology and video surveillance technology are taken advantage of to check existence of cultural relics, their ambient contexts and any abnormal behaviors, respectively. Specifically, the RFID tags attached to cultural relics are checked periodically by comparing the IDs read by RFID readers with what are stored in upper computer, as shown in Figure 9.

The three kinds of sensing techniques are collaboratively used according to the rules {Rule #1, Rule #2, Rule #3} defined below. If the boolean variable *flagMatchAlarm* equals true, then it alarms.

**Rules to determine whether it should alarm.**
**Input:** *flagAllRFIDExist,* a boolean variable representing whether the IDs read from RFID tags attached to all cultural relics match what are preset in upper computer. *True* if they match, *false* if not.*flagAbnormalityDetected*, a boolean variable representing whether abnormal behaviors are detected by video cameras. *True* if detected, *false* if not.*flagAmbientThresholdExceeded,* a boolean variable representing whether ambient parameters exceed the preset threshold that are safe for cultural relics. *True* if exceeded, *false* if not.**Output:** *flagMatchAlarm*, a boolean variable indicating whether it should alarm. *True* if it should, *false* if not.
**Rule #1**
**Begin:** 1:**If** (*flagAmbientThresholdExceeded* == true) **then** 2:{*flagMatchAlarm* = true;} 3:**End If**
**End** 
**Rule #2**
**Begin:** 1:**If** (*flagAllRFIDExist* == false) **then** 2:{*flagMatchAlarm* = true;} 3:**End If**
**End**

**Rule #3**
**Begin:** 1:**If** (*flagAbnormalityDetected* == true && *flagAllRFIDExist* == false) **then** 2:{*flagMatchAlarm* = true;} 3:**Else If** (*flagAbnormalityDetected* == true && *flagAllRFIDExist* == true) **then** 4:{*flagMatchAlarm* = false;} 5:**End If**
**End**

Through this method, detection results from different means can corroborate each other, which can improve the overall accuracy of detection. When an abnormality is detected, the system raises an alarm and sends corresponding messages to appropriate staff immediately to initiate further actions.

### 3.3. Case Study

#### 3.3.1. Application Scenario

**Figure 10:**
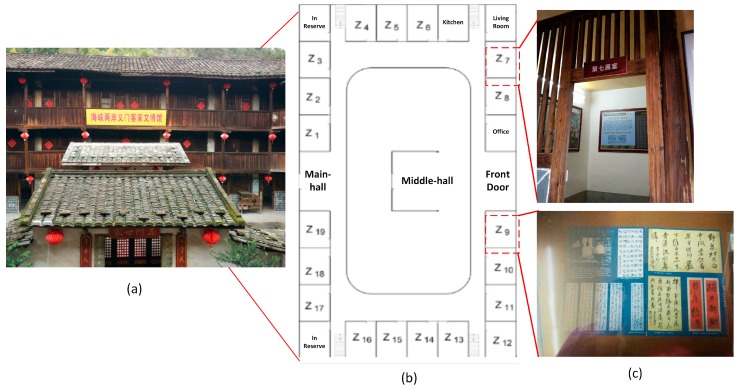
Appearance and layout of the museum in the Yongding District (Fujian Province, China): (**a**) Appearance of the museum, the first floor of which is used to exhibit cultural relics; (**b**) Layout of the first floor of the museum; (**c**) An example of two exhibition regions.

To test the proposed system, we deployed and applied the system to a real-world scenario—a museum in the Yongding District (Longyan, China). As Figure 10 shows, this museum contains one exhibition hall comprising 19 exhibition regions. In each region, a video camera is installed for real-time surveillance. Each cultural relic is attached with an RFID tag and ambient sensors for its identification, real time ambient sensing and preservation. An active RFID reader is deployed at the center of courtyard of the museum, connected to an upper computer via a wired connection. The reader periodically communicates wirelessly with all the RFID tags attached to cultural relics to check their existence and prevent losses.

#### 3.3.2. Results

In this section, we present the results, primarily focusing on three aspects of cultural relic management: initial registration, circulation, and preservation.

**Figure 11:**
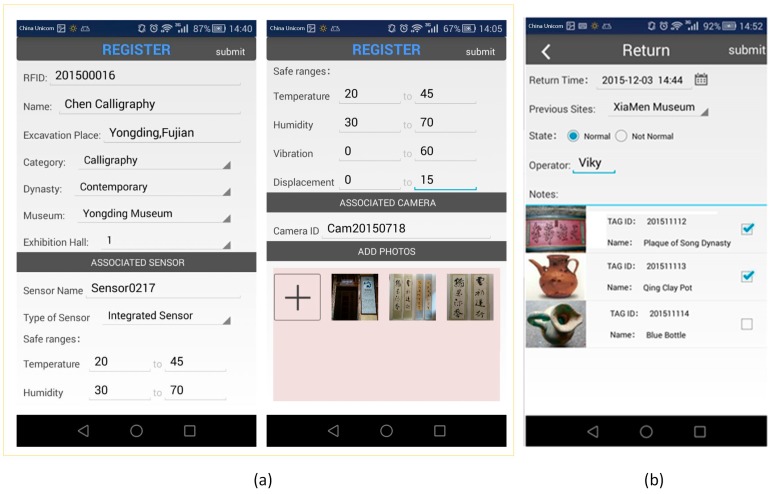
Management results of the application scenario: (**a**) Initial registration of cultural relics; (**b**) Circulation management for cultural relics (the *Return* operation is shown here as an example).

Figure 11a shows the Android-based application GUI used for the initial registration of cultural relics. The ID of the RFID tag attached to the cultural relic is automatically read through an RFID reader. Other data fields, such as the relic's name, excavation place, category, dynasty and so on are manually input by the operators. Ambient sensors and a video camera are associated with the cultural relic at this stage as well.

Figure 11b presents the *Return* operation interface as an example of the circulation management process for cultural relics. The list of cultural relics returned are read automatically by the RFID reader, with *previous site* automatically retrieved from server, *state* manually checked by the operators, *operators* obtained from login info, and *return time* from the system clock.

**Figure 12:**
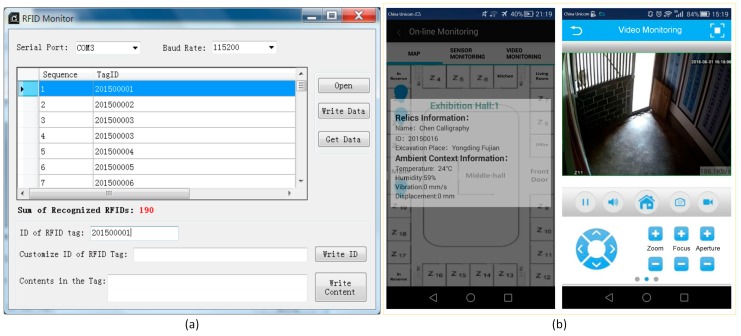
Monitoring results of the application scenario: (**a**) Preservation of cultural relics by checking the existence of RFID tags; (**b**) Preservation of cultural relics through online monitoring using ambient sensors and video cameras.

Figure 12 shows the results of identification of RFID tags by the RFID reader deployed at the center of the museum courtyard and online monitoring of ambient context of cultural relics, using both ambient sensors and video cameras. They work collaboratively to prevent loss of and damage to cultural relics.

## 4. Discussion

### 4.1. RFID-Based Automatic Efficient Identification and Lifecycle Management of Cultural Relics

In the proposed system, RFID is used for identification and tracking of cultural relics, achieving lifecycle management of cultural relics. It’s used in cultural relics’ initial registration, in-out recording and scrap management. In comparison with traditional methods, it provides the following benefits, as summarized in Table 2.

**Figure 13:**
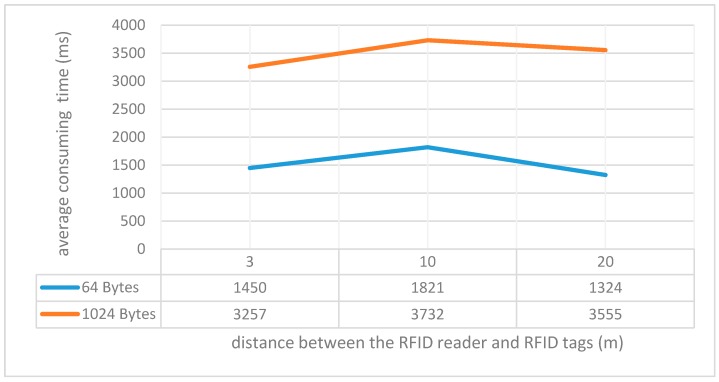
Average time consumed for reading different volumes of data (64 bytes and 1024 bytes in each tag respectively) from all 35 RFID tags simultaneously from different distances (3 m, 10 m, and 20 m). For each distance and volume, 10 tests were performed and the average consuming time was obtained.

Because the RFID tags used in this system support long-range automatic identification which can reach as far as 20 m, cultural relics with RFID tags attached can easily be recognized in a contactless manner. As the RFID tag only stores the unique ID of cultural relics, which usually occupies far less than one thousand bytes of storage space. Therefore, according to our experiments conducted on the RFID tags adopted in the proposed system (as shown in Figure 13), it took 1.45~3.7 s to read all tested tags during the initial registration, circulation or scrap registration of cultural relics, considering that these operations are usually performed within 3~10 m; the stock-take process can be completed in no more than 4 s for all tested cultural relics.

**Figure 14:**
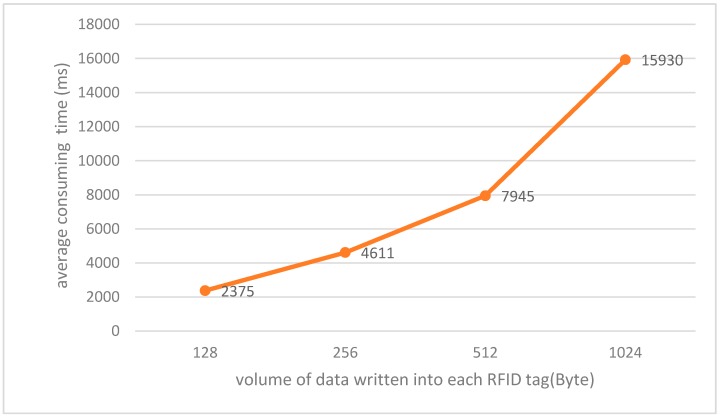
Average time consumed for writing different volumes of data (128 bytes, 256 bytes, 512 bytes and 1024 bytes for each tag respectively) into all 35 RFID tags simultaneously. For each volume, 5 tests were performed and the average consuming time was obtained.

However, as barcodes require that the tag be within line-of-sight and are read one by one, it is obvious that the proposed RFID-based way is far more convenient and efficient than the barcode way, let alone the traditional manual handwritten way. Moreover, as Figure 14 indicates, it took around 2.38~4.61 s to write data into all the tested RFID tags, making initiation of RFID tags or later updates of information stored in these tags easily and efficiently, which however could not be achieved with barcodes for they are read-only and obviously far more efficient than manual handwritten way.

Automation and convenience of operations in the management of cultural relics can be improved to a great extent. Besides, as much information are input from the Android application through simple selection of preset items in the proposed system, errors that may be made by traditional manual means could be reduced significantly.

Moreover, the proposed RFID-based system provides many other advantages, as listed below.

**Figure 15:**
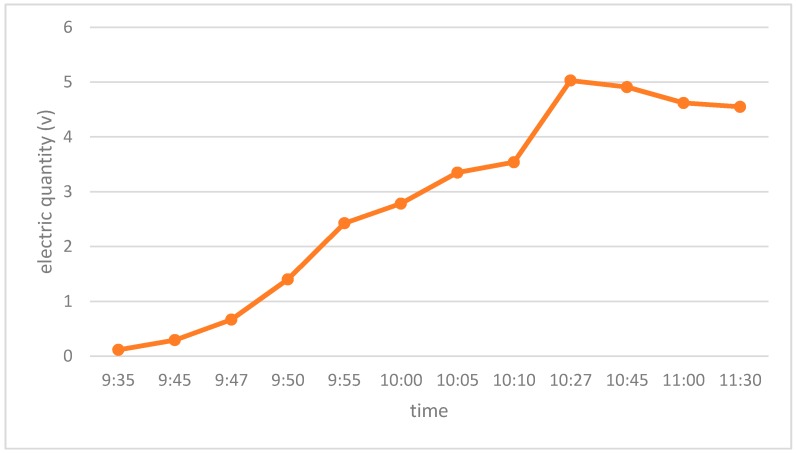
The charge curve of the photovoltaic subtle energy-powered RFID tag adopted in the proposed system. This experiment was conducted in a rainy day. The luminance ranged between 1825 lux and 13,000 lux during the test period of time. The RFID tag was fully charged at 10:27, and the whole charging process took around 50 min.

**Figure 16:**
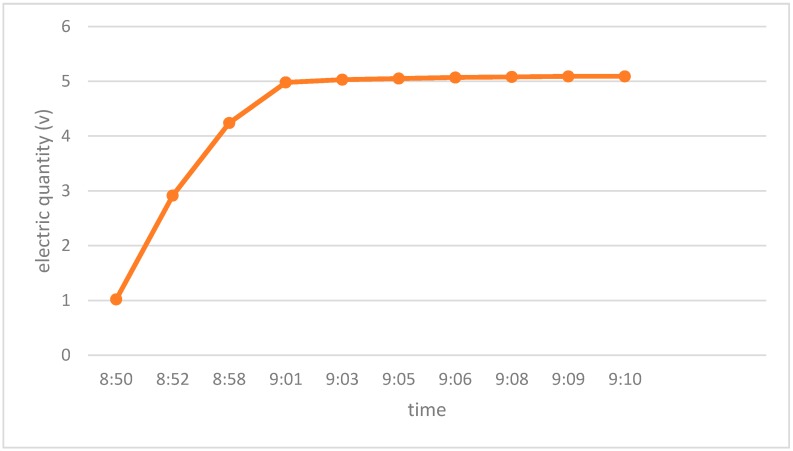
The charge curve of the photovoltaic subtle energy-powered RFID tag adopted in the proposed system. This experiment was conducted in a cloudy day. The luminance ranged between 23,000 lux and 26,000 lux during the test period of time. The RFID tag was fully charged at 09:09, and the whole charging process took around 20 min.

**Figure 17:**
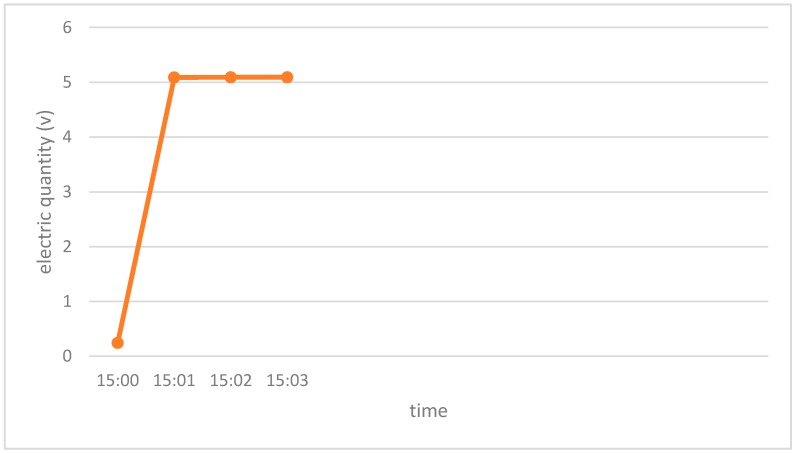
The charge curve of the photovoltaic subtle energy-powered RFID tag adopted in the proposed system. This experiment was conducted in a sunny day. The luminance ranged between 77,000 lux and 85,000 lux during the test period of time. The RFID tag was fully charged at 15:01, and the whole charging process took around 1 min.

(1) The RFID tag used in the proposed system is powered both by photovoltaic subtle energy and by an extra battery. Under most circumstances, energy produced from sunlight is sufficient to power the tag, even though sunlight in the museum may be not so strong. The battery is provided only for the case when the tag has insufficient photovoltaic energy. From the charging curves of the adopted RFID tag under different weather conditions (rainy day, cloudy day and sunny day) with different luminance, as shown in Figure 15, Figure 16 and Figure 17 respectively, the tag can be fully charged in relatively short time even when the sunlight is not so abundant, which makes it fairly suitable for use in an indoor museum environment, and meanwhile much more energy-saving and environmental friendly than traditional battery-based ones.
(2) The RFID tag in our proposed system stores only a unique ID, while the detailed information of cultural relics is stored in the back-end server-side database, reducing the need for storage space in the RFID tag whose storage is limited. Users can obtain details of specific cultural relics easily based on the unique ID and interfaces provided when needed. Moreover, as the frequency of communication between user and back-end server is much lower than that between the RFID tag and the reader, it reduces volume of data transmitted to a great extent and thus reducing the energy consumption during data transmission.
(3) Last but not least, based on the unique ID stored in the RFID tag and the dynamic information stored in the back-end server, users can easily track information concerning the provenance and circulation of cultural relics, i.e., where they originated, where they have been stored and exhibited, where they are now, and where they will be, all of which are highly beneficial for cultural relics management.

### 4.2. Sensor Web-Based Unobtrusive Sensing of Ambient Context of Cultural Relics

Various physical and chemical factors such as light, temperature, relative humidity, and pollutants can affect displayed cultural relics [26]. Thus, monitoring of these ambient contexts is of vital importance to preservation of cultural relics. In our proposed system, air temperature and humidity in each exhibition region is collected at predefined intervals that can be adjusted as needed periodically. Once the monitored data exceeds the predefined threshold that is safe for cultural relics, the system raises an alarm and sends alert information to museum staff immediately who can then take appropriate actions. Through this mechanism, cultural relics are protected from extreme ambient damage and guaranteed to remain in good condition effectively.

### 4.3. Collaborative Monitoring for Comprehensive Safeguarding of Cultural Relics

In traditional cultural relic safeguarding schemes, video surveillance is a common approach that has achieved good results [26,27,28]. RFID technology has also been used to safeguard treasures [18]. Nevertheless, these approaches are typically used individually, whose effects cannot meet the requirements for protecting precious cultural relics. In our proposed system, we take advantage of RFID technology, video surveillance technology, and vibration sensor technology collaboratively to prevent the loss of cultural relics. Each technology has its own mechanism for loss detection that works from different perspectives. They work collaboratively to improve loss detection effects and realize more comprehensive safeguarding of cultural relics than traditional individual way does, contributing to much safer museums.

### 4.4. RFID and Sensor Web-based Architecture for Integrated Management, Monitoring and Preservation of Cultural Relics

Traditional RFID-based systems pay most attention to audits and stock-taking of cultural relics [12,13], while sensor-based client-server systems pay most attention to monitoring of the ambient context of cultural relics [29]. Considering the requirements in modern museums, the proposed system integrates these two technologies, taking advantage of the excellence of RFID in efficient identification and tracking, and the excellence of the Sensor Web in ambient monitoring to realize efficient management and effective preservation of cultural relics. The proposed integrated framework and methods make it more qualified and suitable for use in modern museums than traditional singular technology-based systems.

## 5. Conclusions

RFID- and Sensor Web-related technologies have been one of the main enablers for smart applications in smart cities. They have shown great potential in solving cultural heritage-related problems. For example, they have been used to build a single smart space (S^3^) to improve users’ cultural experiences when visiting cultural heritage [30]. Nevertheless, the simultaneous smart management and preservation of cultural relics still remains a challenge. To help solve this problem, an RFID and Sensor Web-enabled system was designed and implemented. In this system, RFID technologies are adopted for contactless identification, easy and convenient registration and circulation-related management operations of cultural relics, reducing both the time required and errors that could be easily made by manual operations or barcode means. The Sensor Web is also adopted for real-time monitoring of the ambient context of cultural relics, preserving them from damage by physical and chemical factors. Together they record the static metadata (identities and virtual personalities), dynamic status and trace information of cultural relics. Collaboratively used RFID technologies, sensor technologies, and video surveillance technologies are taken advantage of to prevent loss of cultural relics with satisfactory effect. A pilot application in a real-world scenario—a museum located in the Yongding District (Longyan, Fujian Province, China)—successfully demonstrated the feasibility of the proposed system integrating RFID and Sensor Web in helping simultaneously solve the management and preservation problems associated with cultural relics.

Cultural relics are in essence assets. Therefore, the successful experience of adopting RFID and Sensor Web together for management and preservation of cultural relics can also be applied to other domains. For example, we can use RFID for apparatus identification with instructions for manipulation, Sensor Web for ambient monitoring of laboratories, and collaborative safeguard techniques for preservation of expensive apparatus.

Although the proposed system has been tested successfully and has shown its feasibility in efficient management and effective protection of cultural relics, there are still some limitations. For example, the ambient sensors used in our system collect only the ambient air temperature and humidity around cultural relics, which is not enough data for full ambient sensing and preservation. Moreover, while such parameters can be sensed, this system provides no way to adjust them, which leads to a human labor requirement for ambient condition adjustment. Therefore, improving the sensing capabilities and providing the system with ways to automatically adjust ambient context will be the focus of our future work to further improve the preservation of cultural relics, during which big data analysis methods can be taken advantage of. Yet another meaningful direction would be to take 3D indoor Geographical Information Systems (GIS) into consideration to provide better geo-location-based management, tracing and preservation of cultural relics. The further great picture would be that cultural relics are connected to each other, cooperate with each other and can “talk” with each other instantly, which will contribute to smarter and more interactive and alive museums.

## Figures and Tables

**Table 1:** Specifications of context-aware hardware.

Context-Aware Hardware	Sub-Type	Specifications
RFID	tag	Operating frequency: 2.4 GHz Operating distance: 50 m Baud rate: 1 Mbps Power supply: photovoltaic subtle energy and a 1200 mA battery Operating temperature range: −20 °C~70 °C Operating humidity range: 5%~95% (non-condensing) Storage: 1.5 KB
	reader	PDA connectivity means: Bluetooth, USB Bluetooth version: 3.0 Endurance: ≥ 8 h Operating frequency: 2.4 GHz Operating distance: 80 m (depending on tag) Baud rate: 1 Mbps
Ambient sensor	temperature sensor	Temperature sensing range: −40 °C ~85 °C Temperature accuracy: ±0.5 °C
	humidity sensor	Humidity sensing range: 0~100% RH Humidity accuracy: ±3% RH @20%~80% RH, 25 °C
	vibration and displacement sensor	Optional gyroscope full scale range: ±250, ±500, ±1000, ±2000 °/s Optional accelerometer full scale range: ±2, ±4, ±8, ±16 g VDD: 2.5V ± 5%, 3.0V ± 5%, 3.3V ± 5%; VDDIO: 1.8V ± 5%
Video camera	video camera	Sensor type: 1/2.9“, Low Illumination, 2.0 MP CMOS sensor Minimal illumination: 0.01 Lux @ (F1.2, AGC ON) with full color, 0.001 Lux @ (F1.2, AGC ON) with black-and-white; 0 Lux with IR Shutter: 1/50 (1/60)~1/10,000 s Lens: 2.8–12 mm @ F1.4; horizontal field: 74.6°~26.8° Frame rate: 1080P@25fps (PAL); 1080P@30fps (NTSC) Video codec standard: H.264 IR View Distance: 50~60 m Smart alarm: motion detection, video-losing alarm, IP address conflict detection Mobile remote surveillance: iOS and Android support
Mobile terminals	smart mobile phone	Operating System: Android OS 4.4 CPU: 1.7 GHz RAM Storage: 3 GB ROM Storage: 16 GB

**Table 2:** Comparison of the proposed RFID-based system with traditional means.

	Efficiency	Recognition Distance and Environment	Error Rate	Prevention of Loss
Manual	handwritten, one-by-one, very slow	at a short range with cultural relics being touched, mostly during the day with light	high	on-guard, time-consuming, high cost, poor effect
Barcode	automatic, one-by-one, fast	at a short range (0.2~0.76 m), sometimes with cultural relics touched, mostly during the day with light	extremely low	recognized by a fixed reader, at a short range, passive, not safe enough for it’s easy tore off and stained
The proposed RFID-based method	automatic, multiple RFIDs can be read at the same time, very fast (as shown in Figure 13)	contactless, long distance (0.8~20 m), both during the day and night, with or without light.	extremely low	recognized by a fixed/mobile reader, at a long range, active, very safe
